# Editorial

**DOI:** 10.3897/zookeys.856.37027

**Published:** 2019-06-17

**Authors:** Michael Schmitt, Caroline S. Chaboo2, Maurizio Biondi

**Affiliations:** 1 Ernst-Moritz-Arndt-Universität Greifswald, Allgemeine & Systematische Zoologie, Loitzer Str. 26, D-17489 Greifswald, Germany Ernst-Moritz-Arndt-Universität Greifswald Greifswald Germany; 2 Department of Entomology, W-436 Nebraska Hall, University of Nebraska, Lincoln, Nebraska, 68583-0514, USA University of Nebraska Lincoln United States of America; 3 Department of Health, Life and Environmental Sciences, University of L’Aquila, 67100 Coppito-L’Aquila, Italy University of L’Aquila Coppito-L’Aquila Italy

Volume 8 of Research on Chrysomelidae (RoC8) presents again examples of the attractiveness and the diversity of Chrysomelidae (sensu lato) as subjects of scientific research. The seven papers included here cover taxonomy, ecology, faunistics as well as phylogenetics. Four of these papers (Geiser, Gikonyo et al., Salvi et al., Wendorff & Schmitt) are extended versions of talks presented to the Third European Symposium on the Chrysomelidae, held on 5 July, 2018, in Naples, Italy within the frame of the 11^th^ European Congress of Entomology. Maurizio Biondi (L’Aquila, Italy) and Michael Schmitt (Greifswald, Germany) co-organised the Naples meeting and worked together with Caroline S. Chaboo (Lincoln, NE, USA) in the editorial committee for RoC8.

As with the previous RoC volumes, the team at Pensoft Publishers (Sofia, Bulgaria), especially Yordanka Banalieva, did a wonderful job and made the co-operation of editors and publishers a relaxed and rewarding experience. The editors thank our counterparts at Pensoft for this harmonious collaboration. The editors also thank all authors who submitted their high-quality manuscripts and so made this volume another important contribution towards the science of leaf and seed beetles.

The series of symposia on Chrysomelidae will continue with the 10^th^ International Symposium in Helsinki (Finland) in 2020 and the 4^th^ European Symposium on Crete (Greece) in 2022. The proceedings of these symposia along with submitted papers on Chrysomelidae sensu lato will be published in subsequent volumes of Research on Chrysomelidae, as special issues of ZooKeys.

Michael Schmitt, Caroline S. Chaboo, Maurizio Biondi

**Figure:**
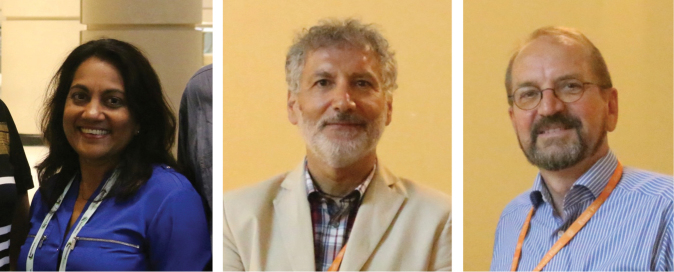
The editors: Caroline S. Chaboo, Maurizio Biondi, Michael Schmitt

